# Antimicrobial Resistance, Virulence Factors, and Genotypes of *Enterococcus faecalis* and *Enterococcus faecium* Clinical Isolates in Northern Japan: Identification of *optrA* in ST480 *E. faecalis*

**DOI:** 10.3390/antibiotics12010108

**Published:** 2023-01-06

**Authors:** Meiji Soe Aung, Noriko Urushibara, Mitsuyo Kawaguchiya, Nobuhide Ohashi, Mina Hirose, Kenji Kudo, Naoyuki Tsukamoto, Masahiko Ito, Nobumichi Kobayashi

**Affiliations:** 1 Department of Hygiene, Sapporo Medical University School of Medicine, Sapporo 060-8556, Japan; 2 Division of Pediatric Dentistry, Department of Oral Growth and Development, School of Dentistry, Health Sciences University of Hokkaido, Ishikari-Tobetsu 061-0293, Japan; 3 Sapporo Mirai Laboratory, Co., Ltd., Sapporo 060-0003, Japan

**Keywords:** *Enterococcus faecalis*, *Enterococcus faecium*, clinical isolates, virulence factor, antibiotic resistance, *optrA*, Japan

## Abstract

*Enterococcus faecalis* and *E. faecium* are the major pathogens causing community- and healthcare-associated infections, with an ability to acquire resistance to multiple antimicrobials. The present study was conducted to determine the prevalence of virulence factors, drug resistance and its genetic determinants, and clonal lineages of *E. faecalis* and *E. faecium* clinical isolates in northern Japan. A total of 480 (426 *E. faecalis* and 54 *E. faecium*) isolates collected over a four-month period were analyzed. Three virulence factors promoting bacterial colonization (*asa1*, *efaA*, and *ace*) were more prevalent among *E. faecalis* (46–59%) than *E. faecium*, while a similar prevalence of enterococcal surface protein gene (*esp*) was found in these species. Between *E. faecalis* and *E. faecium*, an evident difference was noted for resistance to erythromycin, gentamicin, and levofloxacin and its responsible resistance determinants. Oxazolidinone resistance gene *optrA* and phenicol exporter gene *fexA* were identified in an isolate of *E. faecalis* belonging to ST480 and revealed to be located on a cluster similar to those of isolates reported in other Asian countries. The *E. faecalis* isolates analyzed were differentiated into 12 STs, among which ST179 and ST16 of clonal complex (CC) 16 were the major lineage. Nearly all the *E. faecium* isolates were assigned into CC17, which consisted of 10 different sequence types (STs), including a dominant ST17 containing multidrug resistant isolates and ST78 with isolates harboring the hyaluronidase gene (*hyl*). The present study revealed the genetic profiles of *E. faecalis* and *E. faecium* clinical isolates, with the first identification of *optrA* in ST480 *E. faecalis* in Japan.

## 1. Introduction

The genus *Enterococcus* forms the normal flora of the gastrointestinal tract of humans and animals and is associated with an ability to survive and persist in broader environments. Bacterial species of this genus can be opportunistic or nosocomial pathogens causing a wide range of infections in humans, including urinary tract infections, wound infections, intra-abdominal infections, medical device-associated infections, and endocarditis and bloodstream infections [1]. Among *Enterococcus* spp., *E. faecalis* and *E. faecium* are the most prevalent healthcare-associated pathogens worldwide. While they are intrinsically resistant to multiple antimicrobials classes such as cephalosporins, aminoglycosides, lincosamides, and trimethoprim-sulfamethoxazole, enterococcal species have a remarkable ability to acquire new resistance determinants due to the plasticity of their genome [2,3]. Particularly, vancomycin-resistant enterococci (VRE) that acquired *van* gene (e.g., *vanA*) clusters has become one of the major nosocomial bacteria worldwide [2,4].

In recent years, in response to the emergence and rapid spread of VRE, importance of alternative antimicrobial, such as linezolid (LZD), has been increasing [5]. LZD is an oxazolidinone that blocks the bacterial protein synthesis by binding to rRNA on both the 30S and 50S ribosomal subunits [6]. It is one of the last resort antimicrobials for the treatment of infections caused by multidrug-resistant Gram-positive bacteria including VRE and methicillin-resistant *Staphylococcus aureus* (MRSA) [5,7]. However, linezolid resistance has been occurring in *Enterococcus*, which poses a new public health concern [8]. Resistance to linezolid has been known to be linked with mutations in the 23S rRNA V domain and the *rplC*/*rplD* encoding the 50S ribosomal proteins L3/L4, or with the acquisition of *optrA* or *poxtA* encoding for an ATP-binding cassette (ABC)-F protein, or *cfr* encoding 23S rRNA methyltransferase [9]. Among the three resistance genes, *optrA* is considered to be primarily responsible for the LZD-resistant enterococci in isolates from humans through the protection of the bacterial ribosome, a target of oxazolidinones [9,10].

Pathogenesis of enterococci is attributed to a variety of virulence factors, which can be divided into two groups: secreted virulence factors such as cytolysin (*cylA*), gelatinase (*gelE*), and hyaluronidase (*hyl*) and cell surface virulence factors such as aggregation substances (*asa1*), enterococcal surface protein (*esp*), endocarditis antigen (*efaA*), and collagen-binding protein (*ace*), providing important role in biofilm formation, adhesion to host cells, and invasion and facilitation of disease progression [11]. Together with antimicrobial resistance, to monitor the prevalence of virulence factors among *Enterococcus* isolates is significant to understand their clinical impact in a specific region or medical facility, and to consider control measures [12].

In Japan, only limited information is available for the molecular epidemiological characteristics of clinical enterococcal isolates with respect to the prevalence of genotypes, virulence factors, and antimicrobial resistance profile [13,14,15,16]. The prevalence of VRE- and LZD-resistant enterococci appears to be extremely low according to the latest national nosocomial infection surveillance [17]. However, sporadic regional outbreaks of VRE have been reported recently [18,19]. Furthermore, the ratio of oral LZD use to total LZD use increased in the past decade [20], and detection of LZD-resistant *Enterococcus* due to *cfr*/*optrA* or mutation in 23S rRNA in sporadic patients has been reported [21,22,23]. Nevertheless, the prevalence of LZD resistance among clinical isolates remains to be determined.

In the present study, we investigated the prevalence of virulence determinants and antibiotic resistance, and clonal diversity in clinical isolates of *E. faecalis* and *E. faecium* in Hokkaido, the northern main island of Japan. With comprehensive information of the bacteriological profiles of enterococcal isolates, we reported here the identification of an LZD-nonsusceptible *E. faecalis* harboring *optrA*-*fexA*, genotyped as ST480 for the first time in Japanese clinical isolates.

## 2. Results

### 2.1. Bacterial Isolates

A total of 480 enterococci isolates comprising 426 *E. faecalis* and 54 *E. faecium* were analyzed. These isolates were collected consecutively, for a four-month period starting from May 2022 in Sapporo Mirai Laboratory, Co., Ltd., (Sapporo, Japan) where various clinical specimens were submitted from hospitals and clinics in Hokkaido prefecture for microbiological examination. The age range of patients was 8 months to 101 years, while the sex ratio (female/male) was 1.4 (282/198). Sixty-five percent of isolates (311/480) were derived from outpatients. The source of specimens was diverse, with urine being the most common (78.5%, n = 377), followed by vaginal discharge (13.8%, n = 66) and miscellaneous specimens (7.7%, n = 37), including blood (n = 8), pus (n = 7), bile (n = 5), sputum (n = 3), IVH tube (n = 3), wound (n = 2), catheter tip (n = 2), drain fluid (n = 1), urethral stent (n = 1), oral cavity (n = 1), umbilical swab (n = 1), pleural fluid (n = 1), semen (n = 1), and central venous catheter (n = 1). Only one isolate per patient was included in this study.

### 2.2. Prevalence of Virulence Factors

Three virulence factor genes that promote bacterial colonization (*asa1*, *efaA*, and *ace*) were more prevalent among *E. faecalis* (46–59%) than *E. faecium* (Table 1). Enterococcal surface protein gene (*esp*) was virtually the sole cell surface virulence factor detected in *E. faecium*, while showing a similar prevalence to *E. faecalis*. Among extracellularly secreting virulence factors, gelatinase gene (*gelE*) was the most common in *E. faecalis* (58%), followed by the cytolysin gene (*cylA*), whereas in *E. faecium*, only the hyaluronidase gene (*hyl*) was detected at low rate (11%).

### 2.3. Prevalence and Profile of Antimicrobial Resistance and Resistance Determinants

The resistance rates to thirteen antimicrobials, resistance determinants, and their profiles of *E. faecalis* and *E. faecium* clinical isolates are shown in Table 2 and Table 3. The profiles of antimicrobial resistance and prevalence of resistance determinants were considerably different between *E. faecalis* and *E. faecium*. Higher resistance rates and resistance to more antimicrobials were found in *E. faecium*. Levofloxacin (LVX) resistance was identified in nearly all isolates of *E. faecium* (96.3%), in contrast to a significantly lower rate (6.8%) in *E. faecalis*. Although mutations in the quinolone-resistance determining region (QRDR) of both GyrA and ParC were detected in most of the LVX-resistant isolates, mutations in ParC were different between the species. S82R was found in only *E. faecium*, accounting for 46% of LVX-resistant isolates, while almost all *E. faecalis* had the S82I mutation. The majority of *E. faecium* isolates (88.9%) and nearly half of *E. faecalis* isolates showed resistance to erythromycin (ERY), associated with *erm*(B) at a higher prevalence in *E. faecalis*, and *msrC* in only *E. faecium*. High-level gentamicin resistance (GEN-HLR) was noted in 13–19% in both species, while the responsible aminoglycoside modifying enzyme (AME) gene, *aac(6′)-Ie-aph(2″)-Ia* showed a higher detection rate (23%) than GEN-HLR in *E. faecalis*. Among *E. faecalis*, 119 isolates (28%) harbored any one or more of the four AME genes, among which copresence of *aac(6′)-Ie-aph(2″)-Ia* and *aph(3′)-IIIa*, with or without other AME genes was the most commonly found (60.5%, 72/119).

All the isolates were susceptible to vancomycin (VAN), daptomycin (DAP), and teicoplanin (TEC). However, non-susceptibility to LZD (MIC, 4 mg/L) was detected in one isolate of *E. faecalis* (ID, ES443), which harbored *optrA*, representing a detection rate of 0.23% (1/426) in *E. faecalis*. This isolate also had *fexA* and showed resistance to chloramphenicol (CHL) and florfenicol (FFC), (MIC, 16 mg/L and 64 mg/L, respectively), while being susceptible to tedizolid (TDZ) (MIC, <0.25 mg/L).

### 2.4. Characterization of optrA Gene and Its Cluster

**Figure 1:**
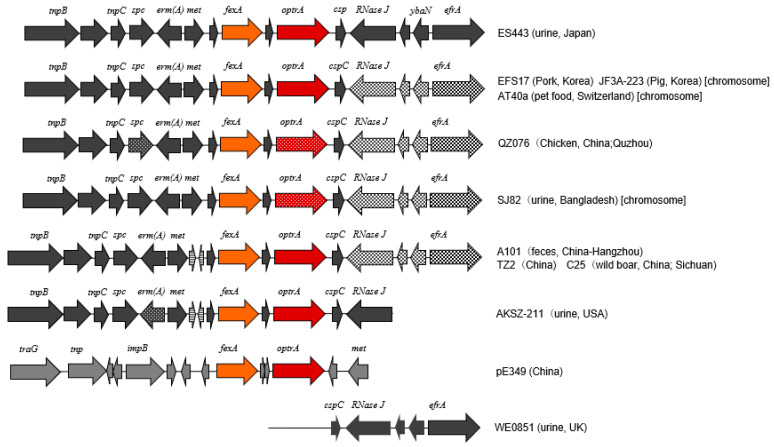
Schematic representation of the genetic background of *optrA* in *E. faecalis* isolate ES443 (uppermost) and the genetic organization or components similar to those of ES443 in other strains reported previously [25] or those available in the GenBank database. Prototype of the *fexA–optrA* cluster in the pE349 of *E. faecalis* strain E349 [24] is shown second from the bottom. Arrows indicate the transcription direction of genes. Different textures in arrows denote divergent sequences of genes. Gene names are shown above arrows, and the strain names are indicated on the right.

Nucleotide sequence of a region surrounding *optrA*, i.e., *optrA* cluster comprising approximately 15 kb, was determined for the LZD-nonsusceptible *E. faecalis* isolate ES443 and compared with the published sequences (Figure 1). The *optrA* gene sequence was identical to that of the prototype gene reported for pE349 [24]. The sequences of this region containing *fexA*-*optrA* and other genes with identical orientations in *E. faecalis* strains were explored by BLAST search. The whole region of ES443 showed the highest identity to that of *E. faecalis* strain EFS17 (from pork in South Korea) (99.86%), JF3A-223 (from pig in South Korea) (99.84%), AT40a (from pet food in Switzerland) (99.83%), QZ076 (from chicken in China) (99.69%), SJ82 (from urine of human in Bangladesh), A101 (from feces of human in China) (99.73%), and so forth. Slight sequence diversity to these strains was found in the downstream region of *optrA* (e.g., *RNase J* and *efrA*). In contrast, *RNase J* gene of ES443 was identical to that of *E. faecalis* strain AKSZ-211 (from environment in China) and WE0851 (from urine of human in the UK).

### 2.5. Genotypes of Isolates with Different Characteristics

Sequence type (ST) based on multilocus sequence typing (MLST) scheme was determined for all the *E. faecium* isolates (n = 54) and selected representative *E. faecalis* isolates (n = 31). *E. faecalis* isolates, which were derived from various specimens, and those that showed different antimicrobial resistance profiles were chosen for MLST (Table 4 and Table 5). Two novel STs of *E. faecalis* (ST1296, ST1305) and three novel STs of *E. faecium* (ST2263, ST2264, ST2267) were identified in the present study.

**Figure 2:**
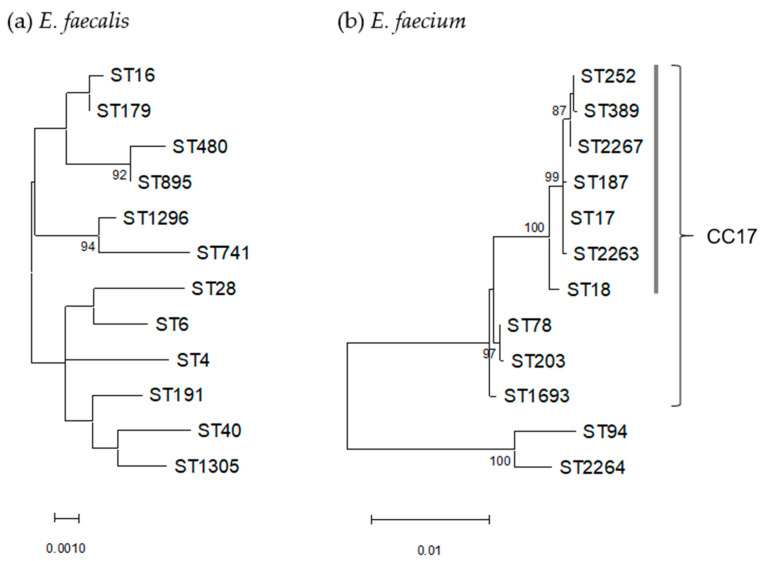
Phylogenetic trees based on concatenated sequences of seven MLST loci of 12 STs each of *E. faecalis* (**a**) and *E. faecium* (**b**). Dendrogram was constructed by maximum-likelihood method with the MEGA11 program and statistically supported by bootstrapping with 1000 replicates, and genetic distances were calculated by the Kimura two-parameter model. Variation scale is shown at the bottom. Percent bootstrap support is indicated by the values at each node (the values <80 are omitted). A cluster of *E. faecium* containing ST17 within CC17 is shown by a vertical bar on the right.

Twelve STs were identified for the *E. faecalis* isolates analyzed. Among them, STs belonging to clonal complex (CC) 16 were the most common (48%; 11 and 4 isolates of ST179 and ST16, respectively), followed by ST1296 (n = 5), ST28 (n = 2), and ST40 (n = 2) (Table 4). The 12 STs of *E. faecalis* were phylogenetically diverse and unrelated, except for CC16 (ST16 and ST179), ST480 and ST895, and ST741 and ST1296 (Figure 2a). Most of ST179 *E. faecalis* isolates (9 among 11 isolates) had 4–6 virulence factors. The prevalence of virulence factors in other STs was variable. Isolates of the prevalent CC16 lineage were generally susceptible to most antimicrobials, except for a few isolates (ES14 and ES116). In contrast, multiple resistance to ERY, LVX, and GEN (high-level) was found in ST4, ST16, and ST1296 isolates. LZD-nonsusceptible isolate ES443 belonged to ST480 and also showed resistance to ERY and LVX, harboring various resistance genes with QRDR mutations and virulence factors *asa1*, *efaA*, and *esp*. In this isolate, *cfr* was not identified, and no mutation was observed in the 23S rRNA gene (V domain) and L3- and L4-encoding genes. ES443 was derived from urine of an outpatient, while no information was available for administration of LZD for treatment of this patient. Although ST480 was identified in only this isolate, among the STs found in the present study, ST895, a single-locus variant of ST480, was found in an isolate.

Nearly all the *E. faecium* (96%, n = 52) were assigned to CC17, which consisted of 10 different STs (ST17, ST18, ST78, ST187, ST203, ST252, ST389, ST1693, ST2263, and ST2267), with ST17 being dominant (59%, n = 32) (Table 5). Among the 10 STs of CC17, two most common types, ST17 and ST78, were genetically distinct, with ST17 forming a main cluster with seven STs (Figure 2b). ST78, a single-locus variant of ST17 (n = 10), contained three isolates positive for the hyaluronidase gene (*hyl*). In contrast, this gene was not detected in ST17 isolates, while *esp* was identified in 66% of ST17. Multiple resistance to ERY, LVX, RIF, and GEN (high level) was found in six isolates belonging to ST17 (four isolates), ST252 and ST2267 (one isolate each). In contrast, isolates of non-CC17 lineage (ST94 and ST2264) were susceptible to most of antimicrobials including LVX, without virulence factors being examined.

## 3. Discussion

The present study revealed the comprehensive status of the antimicrobial resistance, virulence factors, and genotypes of current *E. faecalis* and *E. faecium* clinical isolates in northern Japan. Though the prevalence of individual virulence factors in clinical isolates has not yet been sufficiently studied to date, their incidence in *Enterococcus* species appears to be considerably different depending on origin (human, animal, foodstuff; samples were from infections or healthy individuals) [26,27,28,29,30,31,32,33,34]. The isolates in our study were derived from clinical specimens, mostly from urinary tract infections, and a higher prevalence of *asa1* and *gelE* (approx. 60%) was noted in *E. faecalis*, with other factors, *ace*, *cylA*, *esp*, being detected in 30–50% of isolates. A similarly high rate of *asa1* and *gelE* was described for isolates from food (fish, milk) and animal [29,32,33], and dominance of *gelE* was shown for those from infections in humans [30,31] and ruminants [34]. In contrast, *ace* was ubiquitously distributed to *E. faecalis* from ocular infections in Japan [30] and those from patients, healthy individuals, and the environment in Italy [27]. A difference in the prevalence of *esp* depending on country was also shown [31]. Thus, it is suggested that the prevalence of *ace* and *esp* in *E. faecalis* may be diverse by region as well as infection type, in contrast to the universal distribution of *asa1* and *gelE*. On the other hand, *E. faecium* isolates in our study carried *esp* (approx. 50%) and *hyl* (11%) as the main virulence factors, with the absence of *ace* and *gelE*. Similarly, *esp* was the most prevalent in clinical *E. faecium* isolates in Italy and the UK, while *hyl* was more common in the UK among VAN-resistant isolates [26]. *esp*, which was prevalent in almost half of the clinical isolates of both enterococcal species in our study, is a surface protein associated with biofilm formation through amyloid-like aggregation [35,36], and *hyl* is considered a factor to facilitate intestinal colonization of the bacterial cell involved in the occurrence of infections [37,38]. An increasing prevalence of *esp* and *hyl* in *E. faecium* associated with VAN resistance has been described in European countries [12,39,40], and its global spread is a concern [38]. Therefore, the monitoring of *esp* and *hyl* may be of significance for clinical isolates of *E. faecalis* and *E. faecium*, though the prevalence of *hyl* and VAN resistance is still low in Japan.

The prevalence of antimicrobial resistance observed in the present study seems to be comparable to that from national surveillance [17], without detection of isolates resistant to VAN and TEC. Detection rates of GEN-HLR/*aac(6′)-Ie-aph(2″)-Ia* in *E. faecalis* (13% and 23%, respectively) were lower than our previous study in northern Japan (1997–2007) [13] and Tokyo (2010) [16], suggesting the decrease in GEN-resistance due to infrequent use of aminoglycosides. A higher proportion of *aac(6′)-Ie-aph(2″)-Ia* than GEN-HLR is suggested to be ascribable to the low expression level of *aac(6′)-Ie-aph(2″)-Ia* or the presence of its psedogene [41]. Despite the fact that there have only been a limited numbers of isolates studied, a high prevalence of GEN-HLR and resistance to ERY seems to be persisting in *E. faecium* [13,14,16]. Though *msrC*, which encodes the efflux pump of macrolide, was detected in *E. faecium* at a high rate [14,42], the present study showed a somewhat lower rate (56%), which may suggest that it is not intrinsic to this enterococcal species, as indicated previously [43].

In the present study, a high resistance rate to LVX was noted for *E. faecium* (96%), as observed in our previous study [15], being significantly higher than *E. faecalis* (7%). In both species, mutations in QRDR of both GyrA and ParC were detected in most of the LVX-resistant isolates (90% in *E. faecalis*; 98% in *E. faecium*), while a lower rate was shown in the previous study (72% in *E. faecium*) [15]. The occurrence of mutations in both GyrA and ParC have been described as being related to increased MIC, rather than the presence of a single mutation in either of the proteins [15]. Therefore, the present study indicates further progress of quinolone resistance, particularly in *E. faecium*. In Japan, the proportion of quinolone consumption among all antimicrobial classes is relatively high, with increasing tendency [44], which may be one of the causes spreading quinolone resistance in enterococcus.

In the present study, it was remarkable that an LZD-nonsusceptible *E. faecalis* isolate harboring *optrA* was isolated; *optrA* encodes the ATP-binding cassette (ABC)-F protein, which protects ribosome to confer oxazolidinone resistance [8,45]. Among enterococci, nonsusceptibility to LZD (MIC of 4 mg/L) is prevalent globally at a low rate (<0.38%); a dominant resistance determinant in *E. faecalis* is *optrA* [25]. Nevertheless, a remarkably high prevalence of *optrA*-positive *E. faecalis*/*E. faecium* clinical isolates (1–4%) has been noted in China [8,24,46,47,48,49]. In Japan, the prevalence of *optrA* has not yet been clear, though genomic analysis has been reported for two strains (ST634, ST729) from infected patients [22,23]. In the present study, the prevalence of *optrA*-positive *E. faecalis* could be presumed to be 0.2% (1/426), which may be comparable to Austria (0.2%) [50] and Spain (0.7%) [51]. Genotypes (ST) of *optrA*-positive *E. faecalis* distributed globally have been classified into various STs, including some major STs, i.e., ST16, ST116, ST256, ST476 ST480, ST766, ST775 [24,48,52,53]. ST480, identified in the present study for isolate ES443, has been described as one of the major *optrA*-positive clones in China, Germany, and Ireland [24,54,55], and has also been described in many reports in China and Korea [47,56,57,58,59,60], and European and Latin American countries [10,25,51,52,61]. Our present study is the first identification of *optrA*-positive ST480 *E. faecalis* in Japan, and thus may indicate its global dissemination.

The OptrA amino acid sequence of isolate ES443 was identical to that of the wild type [24,62], and the nucleotide sequence of the *optrA-fexA* cluster was similar to chromosomal elements found in isolates distributed to Asian countries (Figure 1). The structure of the *optrA-fexA* cluster in ES443 was genetically close to those reported in Tn*6674* in chromosome [50,63], while it was distinct from that in plasmid [24,56]. Though the medical history of the patient was not available, ES443 was isolated from the urine of an outpatient as a sole pathogen, showing low MIC to LZD; accordingly, this isolate is not likely to be selected by the use of oxazolidinone. Presumably, other antimicrobials such as LVX or ERY might cause the selective persistence of the *optrA*-harboring strain that might be distributed in the community, because the presence of this gene among healthy populations has been documented [62,64]. Similar views of selection by non-oxazolidinone antibiotics have been described previously for LZD-resistant *E. faecalis* in China [58].

The *E. faecalis* isolates analyzed in the present study belonged to various STs, among which the dominant ST16 and ST179 (CC16) were also common among isolates with GEN-HLR in Japan [16]. A newly identified ST1296 was the second most common, following CC16, and comprised heterologous strains, including an isolate (ES94) with multiple virulence factors and drug resistance. Furthermore, two isolates in our study were identified as ST28, which had multiple virulence factors. This ST had been referred to as a high-risk multidrug resistant strain with a potential public health concern in India [65]. Accordingly, the prevalence of ST1296 and ST28, as well as CC16 *E. faecalis*, should be carefully monitored in Japan.

In contrast, the *E. faecium* isolates in the present study were substantially homogenous, belonging to CC17, which has been known as being responsible for hospital-associated infections, acquiring antimicrobial resistance [66]. Though ST17 was dominant in CC17 in our study, *hyl* was not detected in ST17, but it is commonly present in ST78 (3 positives among 10 isolates). ST78, a single-locus variant of ST17, is described as one of the main genotypes of VRE, particularly those with VanA type in Germany and China [67,68], posing a potential to emerge as a successful clone. Although VanA type VRE is still rare in Japan, attention should be paid to the prevalence and antimicrobial resistance of ST78 *E. faecium*.

The present study revealed the antimicrobial resistance and genetic traits of *E. faecalis* and *E. faecium* that are relevant to potential public health concerns in northern Japan. The obtained findings will contribute to the focus on the important points for further epidemiological surveillance and infection control measures.

## 4. Materials and Methods

### 4.1. Clinical Isolates and Species Identification

Clinical specimens submitted to Sapporo Mirai Laboratory, Co., Ltd. were initially cultured on Sheep Blood Agar plates (Nissui Pharmaceutical, Co., Tokyo, Japan), and occasionally on Columbia CA Sheep Blood Agar plates (Kohjin Bio, Co., Tokyo, Japan) to promote bacterial growth. Species identification was performed by MALDI-TOF mass spectrometry using MALDI Biotyper (BRUKER). All the isolates were confirmed as *E. faecalis* and *E. faecium* by the PCR targeting species-specific sequence of PBP5 genes, as described previously [69]. For some isolates that could not be identified by the PCR, the species was confirmed by the determination of the 16S rRNA gene sequence through direct sequencing with the PCR product amplified by specific primers [70]. Individual isolates were stored in Microbank (Pro-Lab Diagnostics, Richmond Hill, ON, Canada) at −80 °C, and were recovered when they were analyzed.

### 4.2. Antibiotic Susceptibility Testing

Susceptibility to penicillin (PEN), ampicillin (AMP), ampicillin-sulbactam (SAM), imipenem (IPM), high level gentamicin resistance (GEN-HLR), minocycline (MIN), erythromycin (ERY), levofloxacin (LVX), linezolid (LZD), rifampicin (RIF), daptomycin (DAP), teicoplanin (TEC), and vancomycin (VAN) was measured by broth microdilution test, using Dry Plate Eiken DP42 (Eiken, Tokyo, Japan). Antimicrobials and their concentrations (mg/L) used were as follows: PEN (0.12–8), AMP (0.25–8), SAM (1/2–8/16), IPM (0.25–8), GEN-HLR (500), MIN (1–8), ERY (0.25–4), LVX (0.25–4), LZD (0.5–4), RIF (0.5–2), DAP (0.25–4), TEC (0.5–16), VAN (0.5–16). For the LZD non-susceptible isolate (ES443), the MIC of chloramphenicol (CHL), florfenicol (FFC), and tedizolid (TDZ) were determined by the broth microdilution method. Susceptibility/resistance was judged according to the break points mentioned in the CLSI and EUCAST guidelines [71,72]. For CHL and FFC, the MIC breakpoints for susceptibility interpretation was performed as described previously [73].

### 4.3. Detection of Virulence Factors Genes

For all the isolates, the following virulence factor genes were detected by PCR using previously reported primers and conditions [26,27]: aggregation substance (*asa1*), collagen-binding protein (*ace*), virulence factor associated with infective endocarditis (*efaA*), enterococcal surface protein associated with biofilm production (*esp*), gelatinase (*gelE*), cytolysin (*cylA*), and hyaluronidase (*hyl*).

### 4.4. Detection of Drug Resistance Genes

The presence of the following drug resistance genes was examined by uniplex or multiplex PCR assays by primers and conditions, as described previously [41,74,75]: beta-lactamase gene, *blaZ*; aminoglycoside modifying enzymes (AME) genes, *aac(6′)-Ie-aph(2″)-Ia*, *aph(3′)-IIIa*, *ant(6)-Ia*, *ant(4′)-Ia*, *aph(2″)-Id/Ie*, and *ant(9)-Ia*; macrolide resistance genes, *erm*(A), *erm*(B), *erm*(C), *erm*(T), *msr*A, *msr*B, and *msr*C; lincosamide resistance genes, *lnuA*, *lnuB*, *lsaA*, and *mefA/E*; vancomycin resistance genes, *vanA*, *vanB*, *vanD*, and *vanM*; oxazolidinone and phenicol resistance gene, *optrA*, *poxtA*, and *cfr*; and the phenicol exporter gene, *fexA*. Nucleotide sequences of the quinolone resistance-determining region (QRDR) of GyrA and ParC were determined by PCR and direct sequencing to detect mutations [15].

### 4.5. Genetic Determinants of Oxazolidinone Resistance Isolate

One isolate (ES443) exhibiting non-susceptibility to linezolid (MIC = 4 mg/L) was further analyzed for the mutation in 23S rRNA and L3- and L4-encoding genes, as described previously [41]. The nucleotide sequence of the *fexA–optrA* gene cluster was determined by PCR and direct sequencing using the BigDye Terminator v3.1 Cycle Sequencing Kit (Applied Biosystems, Foster City, CA, USA) on an automated DNA sequencer (ABI PRISM 3100). The primers used for sequencing are shown in Table S1 (Supplementary Materials). The multiple alignment of nucleotide/amino acid sequences determined in the present study and those retrieved from the GenBank database was performed by Clustal Omega program (https://www.ebi.ac.uk/Tools/msa/clustalo/, accessed on 10 December 2022), which was also used for the calculation of sequence identity.

### 4.6. Multilocus Sequence Typing (MLST), Phylogenetic Analysis

For all the *E. faecium* isolates (n = 54) and selected *E. faecalis* isolates (n = 31) having different drug resistance profiles and derived from various specimen, the sequence type (ST) based on the MLST schemes [76,77] were identified using the web-based genotyping tool PubMLST (https://pubmlst.org/efaecium/, accessed on 31 October 2022) and (https://pubmlst.org/efaecalis/, accessed on 31 October 2022), respectively. The MLST data were further assigned to the clonal complex (CC) by BURST analysis available in the PubMLST website. To analyze the genetic relatedness of STs identified for *E. faecalis* and *E. faecium*, MEGA11 software (https://megasoftware.net/home, accessed on 28 December 2022) was used to construct the phylogenetic dendrograms of concatenated sequences of seven MLST loci.

### 4.7. GenBank Accession Number

The nucleotide sequences of a genetic cluster, including *fexA–optrA*, was deposited in the GenBank database under the accession number OP795985.

### 4.8. Statistical Analysis

The difference in the prevalence of virulence factors and antimicrobial resistance/resistance determinants between *E. faecalis* and *E. faecium* was statistically analyzed by Fisher′s exact test using the js-STAR XR ver.1.1.9 software (https://www.kisnet.or.jp/nappa/software/star/index.htm, accessed on 31 December 2022). A *p*-value < 0.01 was considered statistically significant.

## Figures and Tables

**Table 1:** Prevalence of virulence factors in clinical isolates of *E. faecalis* (n = 426) and *E. faecium* (n = 54).

Virulence Factor Genes	*E. faecalis* n = 426 (%)	*E. faecium* n = 54 (%)
*asa1* (Aggregation substance)	252 (59.2) *	0 (0)
*efaA* (endocarditis antigen)	209 (49.1) *	1 (1.9)
*cylA* (Cytolysin)	147 (34.5) *	0 (0)
*gelE* (Gelatinase)	248 (58.2) *	0 (0)
*esp* (Enterococcal surface protein)	188 (44.1)	26 (48.1)
*ace* (Collagen-binding protein)	197 (46.2) *	0 (0)
*hyl* (Hyaluronidase)	0 (0)	6 (11.1) *

* Significantly higher rate (*p* < 0.01) between two enterococcal species.

**Table 2:** Prevalence of antimicrobial resistance and resistance genes in clinical isolates of *E. faecalis* (n = 426) and *E. faecium* (n = 54).

Tested Antibiotics/Resistance Genes	*E. faecalis* n = 426 (%)	*E. faecium* n = 54 (%)
Penicillin (PEN)	0 (0)	50 (92.6) *^1^
Ampicillin (AMP)	0 (0)	50 (92.6) *^1^
Ampicillin-Sulbactam (SAM)	0 (0)	50 (92.6) *^1^
Imipenem (IPM)	0 (0)	50 (92.6) *^1^
Minocycline (MIN)	27 (6.3)	2 (3.7)
Erythromycin (ERY)	212 (49.8)	48 (88.9) *^1^
Levofloxacin (LVX)	29 (6.8)	52 (96.3) *^1^
High-level resistance to Gentamicin (GEN-HLR)	54 (12.7)	10 (18.5)
Linezolid (LZD)	1 *^2^ (0.2)	0 (0)
Rifampicin (RIF)	54 (12.7)	24 (44.4) *^1^
*erm*(B)	184 (43.2)	21 (38.9)
*msr*C	0 (0)	30 (55.6) *^1^
*aac(6′)-Ie-aph(2″)-Ia*	98 (23.0)	9 (16.7)
*aph(3′)-IIIa*	93 (21.8) *^1^	2 (3.7)
*ant(6)-Ia*	6 (1.4)	0 (0)
*ant(9)-Ia*	1 (0.2)	0 (0)
*optrA*	1 (0.2)	0 (0)
*fexA*	1 (0.2)	0 (0)
*erm*(A), *erm*(C), *erm*(F), *erm*(G), *erm*(Q), *erm*(T), *erm*(X)*, erm*(Y)*, lunA*, *lnuB*, *lsaA*, *mefA/E, msrA*, *msrB, vanA, vanB, vanD, vanM, poxtA, cfr*	0 (0)	0 (0)

All isolates were sensitive to vancomycin, daptomycin, and teicoplanin. *^1^ Significantly higher rate (*p* < 0.01) between two enterococcal species. *^2^ Number of isolate showing non-susceptibility to LZD (MIC, 4 mg/L).

**Table 3:** Profiles of antimicrobial resistance determinants in *E. faecalis* and *E. faecium* showing resistance to erythromycin, gentamicin, and levofloxacin.

Drug Resistance Determinants ^*1^	*E. faecalis*	*E. faecium*
Macrolide resistance gene	n = 212 (%)	n = 48 (%)
*erm*(B) only	184 (86.8) *^2^	18 (37.5)
*erm*(B) + *msr*C	0 (0)	3 (6.2) *^2^
*msr*C only	0 (0)	27 (56.3) *^2^
Aminoglycoside modifying enzyme gene	n = 170 *^3^ (%)	n = 10 (%)
*aac(6′)-Ie-aph(2″)-Ia*	26 (15.3)	8 (80.0) *^2^
*aph(3′)-IIIa*	19 (11.2)	1 (10.0)
*aph(3′)-IIIa, ant(6)-Ia*	2 (1.2)	0 (0)
*aac(6′)-Ie-aph(2″)-Ia+aph(3′)-IIIa*	67 (39.4)	1 (10.0)
*aac(6′)-Ie-aph(2″)-Ia+aph(3′)-IIIa+ant(6)-Ia*	4 (2.4)	0 (0)
*aac(6′)-Ie-aph(2″)-Ia+aph(3′)-IIIa+ant(9)-Ia*	1 (0.6)	0 (0)
Mutation in QRDR (GyrA/ParC)	n = 29 (%)	n = 52 (%)
S84I/S82I	18 (62.1) *^2^	9 (17.3)
S84Y/S82I	7 (24.1)	17 (32.7)
S84I/S82R	0 (0)	21 (40.4) *^2^
S84Y/S82R	0 (0)	2 (3.8)
S84F/S82R	0 (0)	1 (1.9)
E88G/S82I	1 (3.4)	0 (0)
S84Y, E88G/S82I	0 (0)	1 (1.9)
NM/S82I	2 (6.9)	1 (1.9)
NM/E86K	1 (3.4)	0 (0)

*^1^ QRDR, quinolone-resistance determining region; NM, No mutation; GyrA-S84I (serine to isoleucine), S84Y (serine to tyrosine), S84F (serine to phenyl alanine), E88G (glutamic acid to glycine), ParC-S82I (serine to isoleucine), S82R (serine to arginine), E86K (glutamic acid to lysine). *^2^ Significantly higher rate (*p* < 0.01) between two enterococcal species. *^3^ Number of isolates showing gentamicin MIC of ≥8 mg/L.

**Table 4:** Genotypes, virulence factors and antimicrobial resistance profile of selected *E. faecalis* isolates (n = 31).

Isolate ID	Specimen	Age/Sex	Patient Type	Virulence Factors	Antimicrobial Resistance Profile *^1^	Antimicrobial Resistance Genes	QRDR Mutation *^2^		ST (CC)	Allelic Profile
							GyrA	ParC		
ES 9	sputum	79/M	Inpatient	*asa1*, *efaA*, *cylA*, *gelE*, *esp*, *ace*	ERY	*erm*(B)	NM	NM	ST179 (CC16)	5-1-1-3-7-1-6
ES 10	urine	52/F	Outpatient	*asa1, efaA, cylA, esp, ace*	ERY, GEN-HLR	*erm*(B)*, aac(6′)-Ie-aph(2″)-Ia, aph(3′)-IIIa*	NM	NM	ST179 (CC16)	5-1-1-3-7-1-6
ES 12	urine	82/F	Outpatient	*efaA, cylA, gelE*	LVX		NM	S82I	ST741	15-2-3-88-17-15-11
ES 14	urine	28/F	Inpatient	*asa1*, *efaA*, *cylA*, *gelE*, *ace*	ERY, MIN, GEN-HLR	*erm*(B)*, aac(6′)-Ie-aph(2″)-Ia, aph(3′)-IIIa*	NM	NM	ST16 (CC16)	5-1-1-3-7-7-6
ES 16	urine	96/M	Inpatient	*asa1*, *efaA*, *cylA*, *gelE*, *ace*	All susceptible		NM	NM	ST179 (CC16)	5-1-1-3-7-1-6
ES 27	urine	69/M	Outpatient		ERY, LVX	*erm*(B)	S84Y	S82I	ST895 (ST480 SLV *^1^)	5-1-22-22-7-17-6
ES 36	urine	83/F	Outpatient	*asa1, cylA, gelE*	ERY, GEN-HLR	*erm*(B)*, aac(6′)-Ie-aph(2″)-Ia, aph(3′)-IIIa*	NM	NM	ST179 (CC16)	5-1-1-3-7-1-6
ES 86	urine	95/M	Inpatient	*asa1, efaA, cylA, esp, ace*	ERY	*erm*(B)	E88G	S82I	ST16 (CC16)	5-1-1-3-7-7-6
ES 94	urine	86/F	Outpatient	*asa1*, *efaA*, *gelE*, *esp*, *ace*	ERY, LVX, MIN, GEN-HLR	*erm*(B)*, aac(6′)-Ie-aph(2″)-Ia, aph(3′)-IIIa, ant(6)-Ia*	S84I	S82I	ST1296 *^3^	5-2-3-6-17-1-11
ES 101	urine	83/M	Outpatient	*asa1*, *efaA*, *gelE*, *ace*	ERY, LVX	*aph(3′)-IIIa*	NM	E86K	ST179 (CC16)	5-1-1-3-7-1-6
ES 103	pus	82/F	Inpatient		ERY, LVX, GEN-HLR	*erm*(B)*, aac(6′)-Ie-aph(2″)-Ia, aph(3′)-IIIa, ant(6)-Ia*	S84I	S82I	ST4 (CC4)	8-7-7-5-4-4-1
ES 116	venous blood	73/M	Inpatient		ERY, LVX, GEN-HLR	*erm*(B)*, aac(6′)-Ie-aph(2″)-Ia, aph(3′)-IIIa*	S84Y	S82I	ST16 (CC16)	5-1-1-3-7-7-6
ES 120	urine	48/F	Outpatient		All susceptible		NM	NM	ST16 (CC16)	5-1-1-3-7-7-6
ES 135	Vaginal discharge	32/F	Outpatient	*asa1*, *efaA*, *cylA*, *gelE*, *esp*, *ace*	ERY	*erm*(B)*, aac(6′)-Ie-aph(2″)-Ia, aph(3′)-IIIa*	NM	NM	ST179 (CC16)	5-1-1-3-7-1-6
ES 141	urine	54/M	Outpatient	*asa1*, *efaA*, *cylA*, *gelE*, *esp*, *ace*	ERY, LVX	*erm*(B)	S84I	S84I	ST1296 *^3^	5-2-3-6-17-1-11
ES 151	urine	39/F	Inpatient	*efaA*, *gelE*, *ace*	ERY	*erm*(B)	NM	NM	ST40 (CC40)	3-6-23-12-9-10-7
ES 153	urine	96/F	Outpatient	*asa1*, *efaA*, *cylA*, *gelE*, *esp*, *ace*	ERY, GEN-HLR	*erm*(B)*, aac(6′)-Ie-aph(2″)-Ia, aph(3′)-IIIa*	NM	NM	ST179 (CC16)	5-1-1-3-7-1-6
ES 172	urine	80/F	Inpatient	*asa1*, *gelE*, *esp*, *ace*	All susceptible		NM	NM	ST40 (CC40)	3-6-23-12-9-10-7
ES 174	urine	11/M	Outpatient	*gelE*, *esp*, *ace*	ERY, GEN-HLR	*erm*(B)*, aac(6′)-Ie-aph(2″)-Ia, aph(3′)-IIIa*	NM	NM	ST179 (CC16)	5-1-1-3-7-1-6
ES 179	urine	80/F	Outpatient	*gelE*, *esp*	LVX		S84I	S82I	ST1296 *^3^	5-2-3-6-17-1-11
ES 180	urine	87/F	Inpatient		ERY, LVX, GEN-HLR	*erm*(B)*, aac(6′)-Ie-aph(2″)-Ia, aph(3′)-IIIa*	S84I	S82I	ST1296 *^3^	5-2-3-6-17-1-11
ES 203	urine	94/F	Inpatient	*asa1*, *efaA*, *gelE*, *esp*, *ace*	LVX		S84I	S82I	ST28 (CC28)	4-4-8-3-8-1-3
ES 218	urine	82/F	Inpatient	*asa1*, *efaA*, *cylA*, *gelE*, *esp*	ERY, LVX	*erm*(B)	S84I	S82I	ST1296 *^3^	5-2-3-6-17-1-11
ES 223	catheter tip	72/M	Inpatient	*asa1*, *efaA*, *cylA*, *gelE*, *esp*, *ace*	ERY	*erm*(B)	NM	NM	ST179 (CC16)	5-1-1-3-7-1-6
ES 236	urine	80/M	Inpatient	*asa1*, *efaA*, *cylA*, *gelE*, *esp*, *ace*	LVX		S84I	S82I	ST28 (CC28)	4-4-8-3-8-1-3
ES 239	urine	68/M	Outpatient	*asa1*, *efaA*, *cylA*	ERY, GEN-HLR	*aac(6′)-Ie-aph(2″)-Ia*	NM	NM	ST6 (CC6)	12-7-3-7-6-1-5
ES 290	urine	94/F	Inpatient	*efaA*	All susceptible		NM	NM	ST1305 *^3^	9-6-11-72-78-1-22
ES 297	Vaginal discharge	44/F	Outpatient	*asa1*, *efaA*, *cylA*, *gelE*, *esp*, *ace*	ERY		NM	NM	ST179 (CC16)	5-1-1-3-7-1-6
ES 334	Vaginal discharge	15/F	Outpatient	*asa1*, *efaA*, *cylA*, *gelE*, *esp*	ERY		NM	NM	ST179 (CC16)	5-1-1-3-7-1-6
ES 360	pus	25/F	Outpatient	*efaA*, *gelE*, *esp*	RIF		NM	NM	ST191	27-1-11-1-21-1-2
ES443	urine	74/F	Outpatient	*asa1*, *efaA*, *esp*	ERY, LVX, LZD, CHL, FFC	*erm*(B), *aac(6′)-Ie-aph(2″)-Ia, aph(3′)-IIIa*,*ant(9)-Ia*, *optrA*, *fexA*	S84Y	S82I	ST480	1-1-22-22-7-17-6

*^1^ Abbreviations, see Table 2. CHL, chloramphenicol; FFC, florfenicol. All the isolates were sensitive to PEN, AMP, SAM, IPM, VAN, DAP, TEC. SLV, single-locus variant. *^2^ QRDR, quinolone-resistance determining region; NM, No mutation. *^3^ novel ST identified in this study.

**Table 5:** Genotypes, virulence factors and antimicrobial resistance profile of all the *E. faecium* isolates (n = 54).

Isolate ID	Specimen	Age/Sex	Patient Type	Virulence Factors	Antimicrobial Resistance Profile *^1^	Antimicrobial Resistance Genes	QRDR Mutation *^2^		ST (CC)	Allelic Profile
							GyrA	ParC		
ES 11	bile	92/M	Inpatient	*esp*	ERY, LVX	*msr*C	S84I	S82R	ST17 (CC17)	1-1-1-1-1-1-1
ES 13	urine	93/F	Inpatient	*esp*	ERY, LVX, RIF	*msr*C	S84I	S82R	ST17 (CC17)	1-1-1-1-1-1-1
ES 17	urine	55/F	Outpatient	*hyl*	ERY, LVX, RIF	*msr*C	S84Y	S82I	ST389 (CC17)	1-5-1-1-1-1-3
ES 31	CVC ^*1^	77/M	Inpatient	*hyl*	ERY, LVX, RIF	*msr*C	S84Y	S82I	ST18 (CC17)	7-1-1-1-5-1-1
ES 32	urine	80/F	Inpatient	*esp*	ERY, LVX, RIF	*msr*C	S84I	S82R	ST17 (CC17)	1-1-1-1-1-1-1
ES 35	urine	88/F	Inpatient	*esp*	ERY, LVX, RIF	*msr*C	S84I	S82R	ST17 (CC17)	1-1-1-1-1-1-1
ES 39	urine	90/M	Inpatient	*esp*	ERY, LVX	*erm*(B)	S84I	S82R	ST17 (CC17)	1-1-1-1-1-1-1
ES 56	urine	80/F	Inpatient	*esp*	ERY, LVX, GEN-HLR, RIF	*aac(6′)-Ie-aph(2″)-Ia*	S84Y	S82I	ST17 (CC17)	1-1-1-1-1-1-1
ES 61	urine	91/F	Inpatient	*esp*	GEN-HLR, RIF	*aac(6′)-Ie-aph(2″)-Ia*	S84Y	S82I	ST17 (CC17)	1-1-1-1-1-1-1
ES 64	urine	90/F	Inpatient	*esp*	ERY, LVX	*erm*(B)	S84I	S82R	ST17 (CC17)	1-1-1-1-1-1-1
ES 66	urine	83/M	Inpatient	*esp*	ERY, LVX	*erm*(B)	S84I	S82R	ST17 (CC17)	1-1-1-1-1-1-1
ES 98	venous blood	77/M	Inpatient	*esp*	ERY, LVX, RIF	*erm*(B)	S84I	S82R	ST2263 *^3^ (CC17)	1-1-1-1-1-1-4
ES 102	urine	85/M	Outpatient	*esp*	ERY, LVX	*msr*C	S84I	S82I	ST78 (CC17)	15-1-1-1-1-1-1
ES 106	urine	82/F	Inpatient		ERY, LVX	*msr*C	S84I	S82I	ST18 (CC17)	7-1-1-1-5-1-1
ES 109	urine	90/M	Inpatient		ERY, LVX, GEN-HLR, RIF	*erm*(B), *aac(6′)-Ie-aph(2″)-Ia*	S84Y	S82I	ST17 (CC17)	1-1-1-1-1-1-1
ES 117	bile	86/F	Inpatient		ERY, LVX	*msr*C	S84I	S82I	ST17 (CC17)	1-1-1-1-1-1-1
ES 132	urine	81/F	Inpatient	*esp*	ERY, LVX	*erm*(B)	S84I	S82I	ST17 (CC17)	1-1-1-1-1-1-1
ES 140	bile	77/M	Inpatient		RIF		NM	NM	ST2264 *^3^	25-15-9-33-10-19-6
ES 144	urine	75/F	Inpatient		ERY, LVX, MIN, RIF	*erm*(B), *msr*C	S84Y	S82I	ST78 (CC17)	15-1-1-1-1-1-1
ES 146	urine	82/M	Inpatient	*esp*	ERY, LVX	*erm*(B)	S84I	S82I	ST17 (CC17)	1-1-1-1-1-1-1
ES 148	urine	85/M	Inpatient	*esp*	ERY, LVX	*erm*(B)	S84I	S82R	ST17 (CC17)	1-1-1-1-1-1-1
ES 150	urine	91/F	Inpatient	*esp*	ERY, LVX, GEN-HLR, RIF	*erm*(B), *aac(6′)-Ie-aph(2″)-Ia*	S84Y	S82I	ST17 (CC17)	1-1-1-1-1-1-1
ES 166	urine	90/F	Inpatient		ERY, LVX, RIF	*erm*(B)	S84Y	S82I	ST78 (CC17)	15-1-1-1-1-1-1
ES 170	venous blood	77/M	Inpatient		ERY, LVX, RIF	*msr*C	S84Y	S82I	ST78 (CC17)	15-1-1-1-1-1-1
ES 171	oral cavity	77/F	Inpatient	*esp*	LVX, GEN-HLR, RIF	*aac(6′)-Ie-aph(2″)-Ia*	S84Y	S82I	ST17 (CC17)	1-1-1-1-1-1-1
ES 195	urine	87/F	Outpatient		ERY, LVX	*erm*(B)	S84I	S82R	ST17 (CC17)	1-1-1-1-1-1-1
ES 196	urine	78/M	Inpatient		LVX		S84Y	S82I	ST17 (CC17)	1-1-1-1-1-1-1
ES 209	bile	83/M	Inpatient		ERY, LVX	*erm*(B)	S84I	S82R	ST17 (CC17)	1-1-1-1-1-1-1
ES 211	urine	92/M	Inpatient		LVX, MIN, GEN-HLR, RIF	*aac(6′)-Ie-aph(2″)-Ia*	S84Y	S82I	ST17 (CC17)	1-1-1-1-1-1-1
ES 215	drain fluid	76/M	Inpatient	*esp*	ERY, LVX, RIF	*erm*(B)	S84I	S82R	ST17 (CC17)	1-1-1-1-1-1-1
ES 217	urine	77/M	Inpatient	*esp*	ERY, LVX, RIF	*erm*(B)	S84I	S82R	ST17 (CC17)	1-1-1-1-1-1-1
ES 225	urine	89/F	Inpatient		ERY, LVX, SXT	*erm*(B)	S84Y, E88G	S82I	ST17 (CC17)	1-1-1-1-1-1-1
ES 231	urine	90/F	Outpatient		ERY, LVX, GEN-HLR, RIF	*msr*C*, aac(6′)-Ie-aph(2″)-Ia*	S84Y	S82I	ST2267 *^3^ (CC17)	1-158-1-1-1-1-1
ES 240	pus	75/F	Inpatient		ERY, LVX, RIF	*erm*(B)	S84I	S82R	ST78 (CC17)	15-1-1-1-1-1-1
ES 246	urine	80/F	Inpatient	*esp*, *hyl*	ERY, LVX	*erm*(B), *msr*C	S84I	S82I	ST78 (CC17)	15-1-1-1-1-1-1
ES 254	urine	89/F	Inpatient		ERY, LVX, GEN-HLR, RIF	*erm*(B), *msr*C, *aph(3′)-IIIa*	S84Y	S82I	ST252 (CC17)	1-5-1-1-1-1-1
ES 264	urine	89/F	Inpatient	*esp*	ERY, LVX	*msr*C	S84I	S82R	ST203 (CC17)	15-1-1-1-1-20-1
ES 279	urine	96/F	Inpatient		ERY, LVX	*msr*C	S84I	S82R	ST17 (CC17)	1-1-1-1-1-1-1
ES 280	urine	73/M	Inpatient	*hyl*	ERY, LVX	*msr*C	S84I	S82I	ST78 (CC17)	15-1-1-1-1-1-1
ES 288	urine	82/F	Inpatient	*esp*	ERY, LVX	*msr*C	S84Y	S82R	ST17 (CC17)	1-1-1-1-1-1-1
ES 289	urine	83/F	Inpatient		ERY, LVX	*msr*C	S84Y	S82I	ST78 (CC17)	15-1-1-1-1-1-1
ES 302	urine	74/M	Inpatient	*esp*	ERY, LVX	*msr*C	S84Y	S82R	ST17 (CC17)	1-1-1-1-1-1-1
ES 303	IVH tube	92/F	Inpatient	*esp*	ERY, LVX	*msr*C	S84I	S82R	ST17 (CC17)	1-1-1-1-1-1-1
ES 319	urine	63/M	Inpatient		ERY, LVX	*msr*C	S84Y	S82I	ST1693 (CC17)	9-1-1-1-5-7-1
ES 339	urine	77/F	Inpatient	*esp*	ERY, LVX	*msr*C	S84I	S82R	ST17 (CC17)	1-1-1-1-1-1-1
ES 348	urine	92/F	Inpatient		ERY, LVX, RIF	*msr*C	S84I	S82R	ST17 (CC17)	1-1-1-1-1-1-1
ES 359	urine	50/F	Outpatient		RIF		NM	NM	ST94 (CC94)	13-8-8-8-6-10-6
ES 393	urine	77/F	Inpatient		ERY, LVX	*msr*C	S84I	S82I	ST78 (CC17)	15-1-1-1-1-1-1
ES 407	urine	78/M	Inpatient	*esp*	ERY, LVX	*msr*C	S84F	S82R	ST187 (CC17)	31-1-1-1-1-1-1
ES 410	urine	86/F	Inpatient	*esp*	ERY, LVX	*msr*C	S84I	S82R	ST17 (CC17)	1-1-1-1-1-1-1
ES 434	urine	91/F	Inpatient	*hyl*	ERY, LVX	*erm*(B)	S84I	S82I	ST18 (CC17)	7-1-1-1-5-1-1
ES 421	urine	90/M	Inpatient	*efaA*	ERY, LVX, GEN-HLR	*erm*(B)*, aac(6′)-Ie-aph(2″)-Ia*	NM	S82I	ST17 (CC17)	1-1-1-1-1-1-1
ES 441	urine	75/M	Inpatient		ERY, LVX, RIF	*msr*C	S84Y	S82I	ST17 (CC17)	1-1-1-1-1-1-1
ES 450	urine	78/M	Outpatient	*hyl*	ERY, LVX, GEN-HLR	*msrC, aac(6′)-Ie-aph(2″)-Ia, aph(3′)-IIIa*	S84I	S82I	ST78 (CC17)	15-1-1-1-1-1-1

*^1^ Abbreviations, see Table 2. CVC, central venous catheter. All were sensitive to LZD, VAN, DAP, TEC. All showed resistance to PEN, AMP, SAM, IPM except for four isolates (ES140, ES150, ES215, ES359). *^2^ QRDR, quinolone-resistance determining region; NM, No mutation. *^3^ novel ST identified in this study.

## Data Availability

Not applicable.

## Supplementary Materials

The following supporting information can be downloaded at: https://www.mdpi.com/article/10.3390/antibiotics12010108/s1, Table S1: Primers used for sequencing of *optrA-fexA* cluster identified in this study.
